# Postoperative bleeding complicated by hemophilia A following anal fistula surgery: A case report

**DOI:** 10.1097/MD.0000000000044962

**Published:** 2025-10-03

**Authors:** Xue Chen, Rui Tian, Ze Chen, Longfang Quan, Shaosheng Bei

**Affiliations:** a Department of Proctology, Xiyuan Hospital, China Academy of Chinese Medical Sciences, Beijing, China.

**Keywords:** anal fistula surgery, hemophilia A, postoperative hemorrhage

## Abstract

**Rationale:**

Postoperative hemorrhage is a known complication of anal fistula surgery, but underlying coagulation deficiencies like hemophilia can be a rare and potentially severe cause. In this report, we present a case of recurrent postoperative bleeding after anal fistula surgery caused by hemophilia A.

**Patient concerns:**

This article reports a 48-year-old Chinese male patient who developed repeated wound bleeding and subcutaneous hematoma in the perianal and scrotal regions after high anal fistula incision and seton placement.

**Diagnoses:**

Coagulation studies revealed a significantly prolonged activated partial thromboplastin time of 58.10 seconds. The activated partial thromboplastin time normalized (31.00 seconds) after correction via mixing test. Further coagulation factor activity assays demonstrated a markedly reduced factor VIII level of 2.40 %, leading to a definitive diagnosis of Hemophilia A.

**Interventions:**

Initial hemostatic measures included local compression, suturing, and intravenous hemostatic agents, which proved ineffective. The patient then received fresh frozen plasma transfusions (total 1600 mL); however, no significant improvement was observed. He was subsequently transferred to a specialized center for Factor VIII replacement therapy.

**Outcomes:**

The patient’s wound bleeding gradually ceased. At the 17-day follow-up, the wound was nearly healed with no further bleeding episodes observed.

**Lessons:**

Hemophilia is a risk factor for postoperative bleeding after anal fistula surgery. In patients with moderate or mild hemophilia, symptoms may be subtle, typically only manifesting after trauma or surgery. Therefore, it is crucial to inquire about the patient’s bleeding history and monitor blood test results preoperatively.

## 1. Introduction

Anal fistula is one of the most common diseases in the field of colorectal and anal surgery. Clinically, it is primarily characterized by pain, swelling, and the discharge of blood or pus. Most cases result from the rupture or drainage of perianal or rectal abscesses. Due to the complex and varied pathological changes associated with anal fistulas, the clinical outcomes for some patients may not be favorable. In adults, anal fistulas cannot heal spontaneously without therapeutic intervention. Surgery is the primary treatment approach, and high-level and complex anal fistulas are among the most challenging conditions encountered in colorectal surgery.^[1]^ The anatomical specificity of the anal region presents challenges to postoperative recovery following anal fistula surgery. Potential clinical complications include infection, pain, urinary difficulties, and bleeding. The anorectal region has a characteristic dense vascular network and rich blood circulation. In some cases, the postoperative wound after anal fistula surgery may be large, deep, and open. Additionally, repeated stimulation from suture placement and dressing changes further increases the risk of postoperative bleeding.

Hemophilia is an X-linked recessive genetic disorder caused by a deficiency or reduced activity of functional plasma coagulation factors VIII or IX. It may be inherited or result from a spontaneous mutation.^[2]^ Clinically, bleeding is the primary manifestation and can occur in any part of the body. Hemorrhage in joints and muscles is common, but bleeding may also occur in the brain, gastrointestinal tract, and pharyngeal region. In severe cases, it can be life-threatening. Hemophilia is classified into different severity levels based on the activity of functional coagulation factors. A factor activity level of 5% to 40 % is considered mild, 1% to 5% is moderate, and <1 % is severe.^[3]^ Most patients with hemophilia exhibit bleeding symptoms during infancy or childhood; however, in mild cases, the diagnosis may be delayed.^[4]^

The association between hemophilia and postoperative bleeding following anal fistula surgery is rarely discussed in clinical practice, with only a few case reports available. Therefore, the aim of this article is to confirm the close relationship between coagulation dysfunction and postoperative bleeding after anal fistula surgery, and to further elaborate on the methods and necessity of preoperative screening for mild to moderate hemophilia.

## 2. Case presentation

This article reports the clinical data of a 48-year-old male patient with an anal fistula, who was treated at Xiyuan Hospital, Chinese Academy of Traditional Chinese Medicine and Western Medicine. The patient had a history of hypertension and intermittently took medication for treatment. He had previously undergone a subtotal gastrectomy due to a gastric ulcer.

Three months ago, the patient developed perianal swelling and pain after consuming spicy food. A palpable mass was noted, but there was no significant pain. After applying ointment externally, the mass subsided. Two weeks ago, following another episode of consuming spicy food, the patient experienced perianal discharge, accompanied by yellowish-white secretion, with significant swelling and pain. The patient subsequently sought medical attention at the hospital. Upon conducting an anal examination, the doctor found a 0.5 cm × 0.5 cm fistula opening approximately 3 cm from the anal margin at the 4 o’clock and 7 o’clock positions. A cord-like structure could be palpated at the 3 o’clock position along the fistula tract, with notable tenderness on palpation.

**Figure 1.**
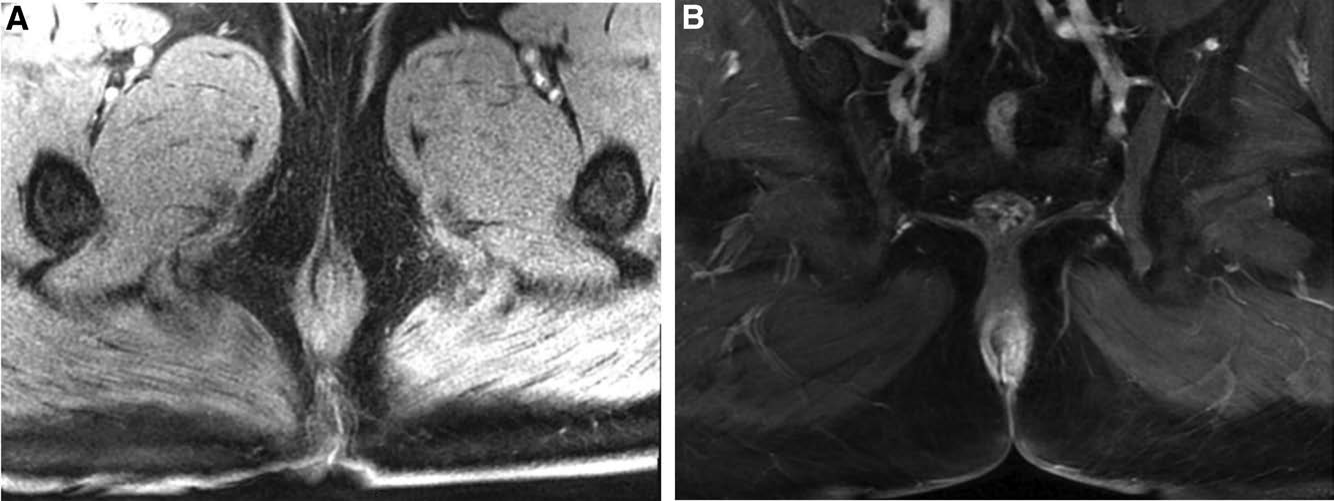
Preoperative MRI of the anal canal with plain scan, diffusion, and dynamic enhancement images: (A) an abnormal signal is observed at the 5 o’clock position (lithotomy position) within the internal anal sphincter, suggestive of the fistula’s internal opening. (B) Approximately 3.6 cm from the anal verge, 2 fistula tracks are seen descending through the intersphincteric branch at the 5 o’clock and 7 o’clock positions. Lithotomy position: the anal canal is conceptualized as the center of a clock face, with the 12 o’clock position corresponding to the patient’s ventral side (anterior) and the 6 o’clock position corresponding to the patient’s dorsal side (posterior). MRI = magnetic resonance imaging.

Coagulation tests showed an Activated Partial Thromboplastin Time (APTT) of 58.10 seconds. The blood routine analysis revealed a mean platelet volume of 9.30 fL and a Platelet-Larger Cell Ratio (P-LCR) of 19.10 %. No significant abnormalities were observed in the infection disease quantitative tests or blood biochemistry. MRI of the anal canal, including plain scan, diffusion, and dynamic enhancement, showed abnormal signals at the 5 o’clock position in the anal sphincter (internal opening), approximately 3.6 cm from the anal margin. Two fistula tracts (at 5 o’clock and 7 o’clock) were visualized, extending downward through the intersphincteric branches, with the external opening at the gluteal skin. The findings are consistent with a low-position intersphincteric complex anal fistula (Fig. 1).

**Figure 2.**
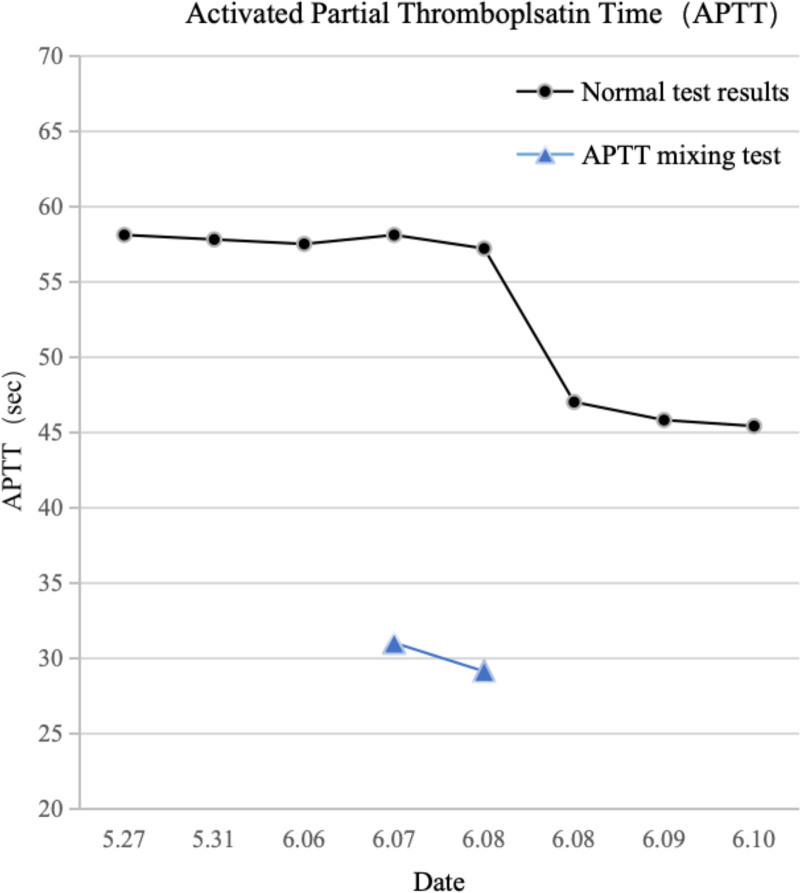
The patient’s APTT values after hospitalization included both normal test results and mixing test results. The normal test results showed that APTT was above the normal range (23.3–35.2 seconds). After the mixing test, APTT decreased to within normal range, indicating a coagulation factor deficiency. APTT = activated partial thromboplastin time.

On hospital day 1, preoperative coagulation tests revealed a prolonged APTT of 58.10 seconds (normal range: 23.3–32.5 seconds) (Fig. 2). Upon further investigation of the medical history, the patient reported having had elevated APTT levels for decades (specific values unknown), with no significant abnormalities detected in previous bone marrow aspiration and related coagulation tests. The patient experienced significant anal swelling and pain, which did not improve with conservative treatment.

On hospital day 2, the patient underwent high anal fistulotomy with seton placement under spinal anesthesia. The patient returned to the ward in stable condition postoperatively with clean wound dressings and no signs of bleeding. Intravenous flurbiprofen axetil was administered for analgesia, and intramuscular metoclopramide hydrochloride was given to prevent nausea and vomiting.

At 10:00 am on postoperative day 1, the patient reported wound oozing, with an estimated blood loss of about 10 mL. Compression with sterile gauze failed to achieve hemostasis. Under local anesthesia with lidocaine injection, figure-of-eight suturing was performed at the 5 o’clock position (lithotomy position) using Polyglactin 910 suture (USP size 000) for hemostasis. Intravenous calcium carbazochrome sulfonate was additionally administered for hemostasis and pain relief.

On postoperative days 2 and 3, the patient continued to experience intermittent wound bleeding. Local compression and suturing were sequentially performed at the 7 o’clock and 4 o’clock positions (lithotomy position) using the same technique. Follow-up complete blood count revealed: RBC 4.19 × 10¹²/L, PDW 9.40 fL, P-LCR 19.10%, and CRP 12.70 mg/L. Coagulation testing showed an APTT of 57.8 seconds (Fig. 2). The patient was additionally treated with intravenous cefoxitin sodium for infection control.

On postoperative days 4 and 5, the patient exhibited punctate oozing from the wound. Thrombin powder was applied topically to the wound surface followed by pressure bandaging on both days.

**Figure 3.**
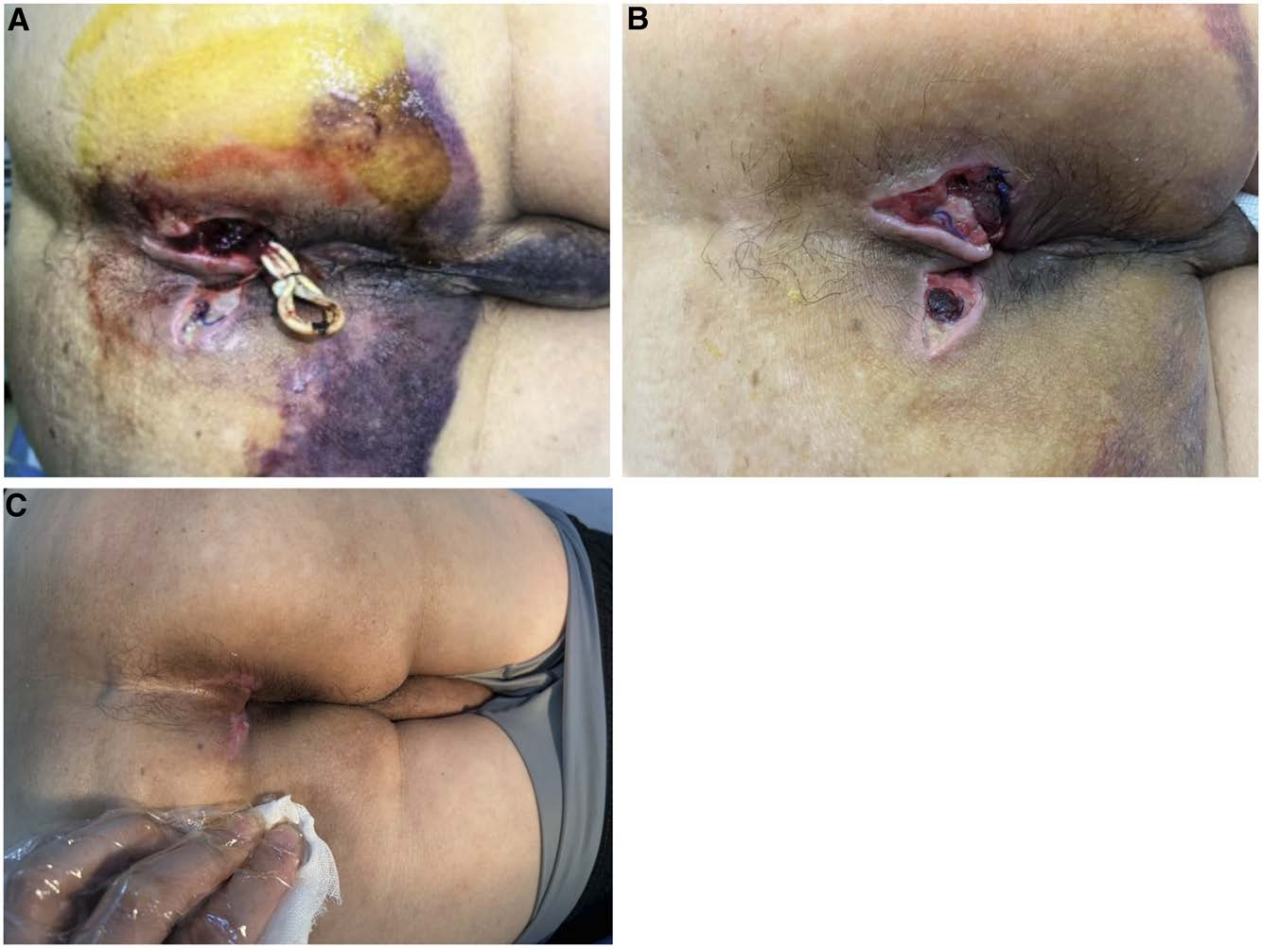
The patient is a 48-year-old male who was admitted for recurrent perianal drainage lasting for 2 weeks. He subsequently underwent high anal fistula incision and seton placement. Postoperative hematoma developed around the wound and scrotum. (A) Day 3 postoperatively, (B) Day 6 postoperatively, (C) Day 17 after discharge.

On postoperative day 6, the patient continues to experience wound oozing, with subcutaneous bleeding observed in the perianal and scrotal regions (Fig. 3A and B). Following hematology consultation, thrombin powder, intramuscular vitamin K1, and intravenous hemocoagulase agkistrodon were administered for hemostasis. However, wound oozing showed no significant improvement. Considering the need for plasma transfusion in addition to compression and intravenous hemostatic agents, the patient declined due to previous ineffective plasma transfusion experience. Further relevant examinations were conducted to investigate the underlying cause.

On postoperative day 9, laboratory results returned: coagulation tests showed D-dimer 1.55 mg/L; APTT 58.10 seconds, FDP 8.88 μg/mL; intrinsic coagulation factor activity assays revealed Factor VIII (FVIII) activity at 2.40%; no significant abnormalities were found in lupus anticoagulant testing. The patient’s intrinsic coagulation factor activity testing revealed a markedly decreased Factor VIII level of 2.40 %, significantly below the normal reference range (50%–150%). Upon further inquiry into the medical history, the patient reported prolonged bleeding after tooth extraction during secondary school, hematoma formation following tongue bites, and subtotal gastrectomy in 2007 due to refractory gastric ulcer bleeding. Postoperatively, he gradually improved following transfusions of red blood cells, platelets (PLTs), and fresh frozen plasma (FFP) (specific volumes unknown).

On postoperative day 10, repeat APTT was 58.10 seconds. Plasma mixing test showed correction of APTT to 31.00 seconds (Fig. 2). Combined with the FVIII level of 2.40% and historical bleeding episodes, Factor VIII deficiency was considered, with a high likelihood of congenital origin. Following consultation with hematologists from a specialized hospital, a supplementary diagnosis of Hemophilia A was made.

On postoperative day 11, after discussion with the patient, 800 mL of type O FFP was administered in 2 transfusions. After the first 400 mL transfusion, APTT was 47.00 seconds, and the plasma mixing test showed APTT correction to 29.10 seconds. Over the next 2 days, 400 mL of plasma was transfused daily, with subsequent APTT values of 45.80 seconds and 45.40 seconds, respectively (Fig. 2). After 3 consecutive days of plasma transfusion totaling 1600 mL, there was no significant improvement in APTT, and the wound continued to exhibit slight oozing with a small amount of purulent discharge. No peripheral skin edema was observed. After comprehensive discussion with the patient’s family, the patient was transferred to another hospital on postoperative day 14 for Factor VIII replacement therapy.

At the 17-day postoperative follow-up in our outpatient clinic, the patient reported gradual cessation of wound oozing following Factor VIII infusion. Physical examination revealed that the wound was nearly healed (Fig. 3C).

## 3. Discussion

Hemophilia is an X-linked recessive bleeding disorder caused by mutations in the genes for coagulation factors, resulting in a deficiency of Factor VIII (FVIII) in Hemophilia A or Factor IX (FIX) in Hemophilia B. The severity of the disease depends on the level of reduction in FVIII or FIX. Hemophilia A and Hemophilia B predominantly affect males, while females are usually carriers of a single mutated gene and typically exhibit only mild symptoms.^[5]^ The prevalence of Hemophilia A in the general population is approximately 1 in 5000, while the prevalence of Hemophilia B is about 1 in 40,000. Hemophilia A tends to be more severe than Hemophilia B in terms of genetic mutation severity and clinical manifestations. For Hemophilia A, approximately 40 % of patients have severe hemophilia, about 10 % have moderate hemophilia, and the remaining 50 % are diagnosed with mild hemophilia.^[5,6]^ Patients with severe hemophilia are at risk of spontaneous, life-threatening bleeding events, while those with moderate or mild hemophilia typically experience abnormal bleeding only following trauma or surgery.^[7]^

The bleeding risk in patients with hemophilia is due to insufficient thrombin generation caused by the deficiency of upstream coagulation factors. Vascular injury following trauma or surgery allows tissue factor to interact with circulating activated coagulation factor VII (FVII), leading to the generation of early thrombin. Thrombin recruits additional enzyme complexes to amplify the thrombin burst, enhancing its strength. In Hemophilia A and Hemophilia B, the deficiencies of FVIII and FIX, respectively, lead to insufficient thrombin generation in the coagulation cascade. This inadequate thrombin burst impairs the ability to form a stable fibrin clot when needed, ultimately resulting in a tendency to bleed.^[5]^ The levels of FVIII and FIX are typically measured using a coagulation function test based on the APTT. In hemophilia patients, prothrombin time (PT) and platelet count remain normal, while APTT is prolonged. If APTT is prolonged, further correction tests are required to clarify the underlying cause and to preliminarily differentiate between coagulation factor deficiencies and coagulation factor inhibitors.^[8]^ In 30 % of patients with mild to moderate Hemophilia A, certain genetic defects can result in FVIII activity measured by the 1-stage assay being higher than that measured by the 2-stage or chromogenic assay, leading to potential misdetection. Additionally, severe Von Willebrand disease can also cause a decrease in FVIII levels, so Von Willebrand disease antigen levels should also be monitored to rule out the possibility of misdiagnosis.^[9]^

The primary treatment for Hemophilia A is replacement therapy, which involves infusing the deficient coagulation factor to achieve hemostasis. FVIII has a half-life of only 8 to 12 hours in the body. While the hemostatic effect is significant, it is not long-lasting. Therefore, appropriate frequencies and doses of coagulation factors must be administered based on the type and severity of the bleeding, with repeated infusions required in the event of re-bleeding.^[10]^ In recent years, gene therapy^[11]^ and bispecific monoclonal antibody therapy^[12]^ have garnered widespread attention, offering more comprehensive treatment and preventive management options for patients with hemophilia. In this case, we initially administered FFP to the patient to replenish coagulation factors. However, 1 mL of FFP contains 1 unit of factor activity, it was difficult to raise the patient’s factor VIII level above the hemostatic threshold of 30 IU/dL through FFP infusion alone without incurring fatal volume overload.^[13]^ Consequently, the postoperative bleeding symptoms showed no significant improvement. It was only after the patient was transferred to a specialized hospital and received factor VIII concentrate replacement therapy that the surgical wound bleeding was effectively controlled and gradually healed.

Hemorrhage is a relatively dangerous and serious complication of anal fistula surgery. Due to the contractile activity of the local anal sphincter muscles, postoperative bleeding at the wound site tends to be concealed and difficult to detect promptly. If the underlying cause is not accurately identified and hemostasis is not effectively achieved, excessive bleeding may occur, potentially leading to anemia or even hypovolemic shock, thereby posing a threat to the patient’s life. In this case, after detecting bleeding at the patient’s wound site, we initially applied pressure, sutured, and administered hemostatic medications via intravenous infusion, but none of these measures showed significant efficacy. During the hemostasis process, we identified several points that were distinctly different from the typical progression of bleeding following standard anal fistula surgery: no obvious arterial or venous bleeding points were identified, with the bleeding primarily characterized by chronic oozing. The amount of bleeding was relatively low, but the frequency of bleeding was high, and the duration was prolonged. Temporary pressure bandaging provided relief, but bleeding recurred once the external pressure was removed. We considered the possibility that the bleeding was caused by the surgery and subsequently conducted a thorough investigation. It was found that the patient had abnormal blood coagulation, with the endogenous clotting factor activity test revealing that factor VIII was 2.40%, significantly lower than the normal level. After consulting with specialists, the final diagnosis of “Hemophilia A” was confirmed.

## 4. Patient perspective

The patient expressed that obtaining a definitive diagnosis of Hemophilia A provided clear medical explanation for his condition. He reported previous episodes of prolonged bleeding after tooth extraction and hematoma formation following tongue bites. In 2007, he underwent subtotal gastrectomy due to gastric ulcer bleeding, but no definitive cause for his bleeding tendency had been established until now. When plasma transfusion was initially recommended, the patient declined based on previous treatment experience. He stated: “I received plasma transfusion for gastric ulcer bleeding in 2007, but the effect was unsatisfactory. Therefore, I have reservations about receiving plasma transfusion again.” The postoperative recurrent wound oozing caused significant concern. The patient described: “I felt helpless and fearful every time the wound started bleeding again.” After being transferred to a specialized center and receiving Factor VIII replacement therapy, the bleeding was effectively controlled.

## 5. Conclusions

The patient in this case had no prior diagnosis of hemophilia, but preoperative coagulation tests revealed a significantly prolonged APTT (58.10 seconds) along with a clear history of bleeding manifestations. Although the patient reported long-standing APTT abnormalities and had previously undergone specialized hospital evaluations that ruled out hemophilia, detailed postoperative inquiry revealed that the patient had received a transfusion of 10,000 cc of blood due to gastrointestinal ulcer bleeding prior to those specialized tests. The exogenous coagulation factors in the transfused blood may have previously complicated the previous diagnostic process for hemophilia. This case underscores the critical importance of comprehensive bleeding history collection and precise coagulation assessment preoperatively. Even in the absence of a confirmed hemophilia diagnosis, the combination of abnormal coagulation parameters and a clinical history of bleeding should raise strong suspicion for underlying coagulopathy. Further targeted investigations, such as coagulation factor activity assays, must be conducted to mitigate perioperative bleeding risks.

## Acknowledgments

We thank the efforts and contributions of the reported patients and all the clinical staff in this study.

## Author contributions

**Investigation:** Ze Chen, Longfang Quan.

**Methodology:** Rui Tian, Longfang Quan.

**Writing – review & editing:** Xue Chen, Shaosheng Bei.
